# Multi-channel functional near-infrared spectroscopy for real-time monitoring during carotid artery stenting: a case series

**DOI:** 10.3389/fnins.2025.1728346

**Published:** 2026-01-14

**Authors:** Xin Gu, Yun-Hsuan Chen, Anwen Shao, Congguo Yin, Mohamad Sawan, Keqin Liu

**Affiliations:** 1 Department of Neurology, Affiliated Hangzhou First People’s Hospital, School of Medicine, Westlake University, Hangzhou, China; 2 CenBRAIN Neurotech Center of Excellence, School of Engineering, Westlake University, Hangzhou, China; 3 Institute of Advanced Technology, Westlake Institute for Advanced Study, Hangzhou, China; 4 Department of Neurosurgery, The Second Affiliated Hospital, School of Medicine, Zhejiang University, Hangzhou, China

**Keywords:** blood–brain barrier, carotid artery stenting, case report, cerebral oxygenation monitoring, functional near-infrared spectroscopy, hemodynamic complications

## Abstract

Functional near-infrared spectroscopy (fNIRS) offers a non-invasive method to monitor cerebral oxygenation and hemodynamic changes in real time. Transient hemodynamic instability during carotid artery stenting (CAS) may compromise blood–brain barrier (BBB) integrity and cerebral homeostasis, highlighting the need for sensitive intraoperative monitoring. Compared with traditional dual-channel NIRS systems limited to the frontal region, multi-channel fNIRS enables spatially resolved assessment of cortical hemodynamics and may provide a valuable adjunct for cerebroprotection research. We retrospectively analyzed five consecutive patients who underwent CAS under local anesthesia, with continuous multi-channel fNIRS monitoring before, during, and after the procedure. Forty-five cortical channels covering bilateral anterior circulation were recorded. Changes in oxyhemoglobin concentration (*Δ*[OxyHb]) and its standard deviation around key procedural events were analyzed to evaluate cortical oxygenation dynamics. All five procedures were technically successful, reducing residual stenosis to less than 30%. fNIRS consistently revealed increased cortical oxygenation following angioplasty and stent deployment, indicating improved cerebral perfusion. In four patients, transient fluctuations in cortical oxygenation corresponded to perioperative hemodynamic instability, such as bradycardia or hypotension induced by carotid sinus reflex. In one patient, marked declines in cortical oxygenation preceded transient neurological deficits, with recovery parallel to blood pressure normalization. These findings suggest that multi-channel fNIRS can sensitively capture both global and regional alterations in cerebral oxygenation during CAS, providing real-time insight into perfusion dynamics potentially linked to BBB function and cerebroprotection. Future studies integrating fNIRS with BBB-targeted markers may help refine intraoperative neuroprotection strategies in stroke and vascular interventions.

## 1 Introduction

Carotid artery stenting (CAS) is one of the main therapeutic approaches for carotid artery stenosis, and its safety and efficacy have been generally recognized (Reiff et al., 2019; Bae et al., 2015; White et al., 2022). Nevertheless, CAS may be complicated by embolic events, hyperperfusion syndrome, contrast-induced encephalopathy, bradycardia, hypotension, and vascular access–related hemorrhage (Nicosia et al., 2010). Among these, bradycardia and hypotension are often triggered by carotid sinus reflex activation and can significantly increase the risk of perioperative neurological events (Gupta et al., 2006; Howell et al., 2002). Although performing CAS under general anesthesia can reduce movement-related hemodynamic fluctuations, it simultaneously eliminates patients’ clinical feedback, hindering timely recognition of perioperative hypoperfusion or embolic complications (Gekka et al., 2021). This underscores the importance of reliable intraoperative neuromonitoring.

Functional near-infrared spectroscopy (fNIRS) is an emerging non-invasive neuroimaging technique that detects cortical oxygenation and hemodynamic changes associated with neuronal activation (Ferrari and Quaresima, 2012). Compared with earlier near-infrared spectroscopy (NIRS) systems, fNIRS provides higher spatial resolution, multi-channel recording capability, and improved adaptability for functional mapping, enabling real-time assessment of cortical oxygenation dynamics. To date, fNIRS has been widely applied in cognitive neuroscience, neonatology, and psychiatry (Pinti et al., 2020; Peng and Hou, 2021; Kumar et al., 2017). However, despite its broad use in these domains, its application during carotid interventions remains extremely limited. Most intraoperative optical monitoring studies in carotid surgery have relied on traditional dual-channel NIRS—primarily during carotid endarterectomy to detect clamp-induced ischemia or postoperative hyperperfusion—while reports employing multi-channel fNIRS during CAS are scarce, and no standardized methodology has been established. Thus, systematic evidence supporting the intraoperative use of fNIRS in CAS remains lacking (di Biase et al., 2022). In parallel, recent experimental and preliminary clinical studies have shown that optical hemodynamic signatures may reflect vascular instability and endothelial stress associated with blood–brain barrier (BBB) dysfunction during ischemia–reperfusion (Milej et al., 2017; Forcione et al., 2020). Although these findings are indirect, they raise the possibility that real-time fNIRS may capture cortical responses related to BBB vulnerability during carotid revascularization.

In this study, we present a case series utilizing multi-channel fNIRS to continuously monitor cortical hemodynamics before, during, and after CAS, aiming to evaluate its feasibility and its effectiveness in detecting hemodynamic disturbances in real time. By integrating multi-channel optical monitoring with cerebrovascular pathophysiology, this work seeks to explore whether fNIRS can provide complementary information about intraoperative cortical responses that are not captured by conventional monitoring modalities.

## 2 Materials and methods

### 2.1 Patients

Five consecutive patients who underwent carotid artery stenting (CAS) under local anesthesia at our institution between March 2023 to May 2023 were retrospectively included. Inclusion criteria were: (1) age ≥18 years; (2) symptomatic or asymptomatic carotid stenosis requiring CAS based on guideline-indicated criteria; (3) ability to undergo continuous multi-channel fNIRS monitoring throughout the procedure; and (4) availability of complete clinical, angiographic, and intraoperative records. Exclusion criteria included conversion to general anesthesia, incomplete fNIRS acquisition due to technical issues, emergent CAS where proper optode placement was not feasible, scalp conditions precluding sensor attachment, or refusal of data use.

Clinical data, imaging findings, treatment details, and outcomes of five patients who underwent CAS were retrospectively reviewed. Among them, four patients had severe stenosis at the origin of the left internal carotid artery, and one patient had severe stenosis on the right side. All patients received dual antiplatelet therapy prior to the procedure. Their demographic characteristics, presenting symptoms, and disease severity are summarized in Table 1. Preoperative cardiopulmonary assessments were performed to ensure surgical eligibility, and contraindications were excluded. Hypotension was defined as a systolic blood pressure reduction of >40% from baseline, and bradycardia as a heart rate reduction of >20% from baseline. All patient data were de-identified.

**Table 1:** Basic information of all patients.

Patient	1	2	3	4	5
Age (years)	76	67	74	82	71
Sex	Male	Male	Male	Male	Male
CYP2C19	*1/*1	*1/*1	*1/*2	*1/*3	*1/*1
Drug preparation	aspirin 100 mg QD + clopidogrel 75 mg QD	aspirin 100 mg QD + clopidogrel 75 mg QD	aspirin 100 mg QD + tigrillo 90 mg QD	aspirin 100 mg QD + clopidogrel 75 mg QD	aspirin 100 mg QD + clopidogrel 75 mg QD
Treated hypertension	Yes	No	Yes	Yes	Yes
Ischemic heart disease	No	No	No	No	Yes
Diabetes mellitus	No	No	No	No	No
Smoking	Yes	No	Yes	No	Yes
Transient ischemic attack	Yes	No	No	No	No
Ischemic stroke	No	Yes	Yes	Yes	Yes
Responsible vessel	LICA-C1	LICA-C1	LICA-C1	LICA-C1	RICA-C1
Ipsilateral stenosis	95%	93%	90%	88%	82%
Remote protection device	Emboshield NAV6 7.2 mm	Emboshield NAV6 7.2 mm	Emboshield NAV6 7.2 mm	Emboshield NAV6 7.2 mm	Spider FX 6.0 mm
Pre-dilated balloon	Viatrac 14 Plus 4.0*30 mm 6 atm	Viatrac 14 Plus 4.0*30 mm 8 atm	Viatrac 14 Plus 4.0*30 mm 6 atm	Viatrac 14 Plus 4.0*30 mm 8 atm	Viatrac 14 Plus 5.0*30 mm 6 atm
Carotid stent	Protégé 10–7*40 mm	Protégé 8–6*40 mm	Protégé 10*40 mm	Protégé 10–7*40 mm	Protégé 8–6*40 mm, Protégé 8*60 mm
Post-dilated balloon	No	No	No	No	Viatrac 14 Plus 4.0*20 mm 6 atm, Viatrac 14 Plus 6.0*20 mm 6 atm
Blood pressure condition	Hypotension	Stable	Hypotension	Stable	Hypotension
Symptomatic nervous system injury	Reduced consciousness, aphasia, diminished muscle strength	Right hemiplegia, part expressive aphasia	None	None	None

“*” denotes CYP2C19 allele nomenclature (e.g., *1, *2, *3); when used for endovascular devices, it serves as a separator between size parameters.

### 2.2 Ethics statement

The studies involving humans were approved by the Ethics Committee of Affiliated Hangzhou First People’s Hospital, School of Medicine, Westlake University (Hangzhou, China). The study was conducted in accordance with the local legislation and institutional requirements. Written informed consent for participation and for publication of anonymized data and images was obtained from all participants in accordance with institutional guidelines.

### 2.3 CAS procedure

All patients underwent standardized CAS under local anesthesia following adequate preoperative preparation. Intraoperative monitoring included fNIRS, non-invasive blood pressure, and peripheral oxygen saturation. Periprocedural anticoagulation was achieved with intravenous unfractionated heparin. All procedures were performed via the right transfemoral approach. An 8 Fr guiding catheter (MACH 1™, Boston Scientific) was advanced into the distal common carotid artery (CCA) using a coaxial technique. Digital subtraction angiography was used to assess stenosis severity and intracranial circulation. A distal embolic protection device (EPD; Emboshield NAV6™, Abbott or Spider FX™, EV3) was deployed in the cervical segment of the internal carotid artery (ICA). Predilatation was performed with an undersized balloon (RX Viatrac 14 Plus, Abbott) before stent implantation. A self-expanding stent (Protégé™ RX, EV3) was then deployed across the lesion, with postdilatation performed if necessary. After stent release, the catheter and EPD were retrieved, and femoral puncture hemostasis was achieved using vascular closure devices (Perclose ProGlide™, Abbott).

### 2.4 fNIRS monitoring and signal preprocessing

Functional near-infrared spectroscopy was recorded using a continuous-wave system (NIRSport2, NIRx Medical Technologies LLC, Glen Head, NY, USA) with dual wavelengths (760/850 nm), a sampling rate of 8.138 Hz, and an estimated penetration depth of 1–2 cm below the scalp. Forty-five long-separation channels (30-mm source-detector distance) were arranged according to the 10–20 system to cover bilateral frontal, temporal, and parietal cortices, with particular emphasis on regions supplied by the middle cerebral artery. Additional channels outside the MCA territory served as references. fNIRS signals were continuously acquired before, during, and after CAS, and all key procedural events—including balloon inflation/deflation and stent deployment/release—were time-stamped.

Raw light-intensity data were preprocessed in nirsLAB. Channels with poor optical coupling or a coefficient of variance ≥20% were excluded. The remaining channels were band-pass filtered between 0.01 and 0.2 Hz to remove cardiac, respiratory, and low-frequency drift components. *Δ*[OxyHb] was derived using the modified Beer–Lambert Law. Because CAS was performed under local anesthesia with mild sedation, head movement was minimal, no abrupt motion spikes were detected during quality inspection, and no additional motion-correction algorithm was applied. Baseline correction was not applied because the goal of this study was to characterize continuous intraoperative fluctuations rather than task-evoked responses.

To quantify rapid hemodynamic variability, the standard deviation (STD) of *Δ*[OxyHb] was computed within a ± 6-s window around each procedural timestamp. This metric reflects short-term instability associated with hemodynamic perturbations. These STD elevations were interpreted as physiologic fluctuations rather than motion artifacts, based on visual inspection of the raw light-intensity traces.

Short-separation channels were not included in this system; therefore, extracranial components could not be directly regressed out. This limitation is acknowledged in the Discussion. Nonetheless, the likelihood of substantial extracranial contamination was reduced by preserved SpO₂ (>95%), minimal blood loss, and the bilateral symmetry of temporal patterns observed across homologous channels. Future studies will incorporate short-channel regression and additional physiological regressors to further isolate cortical signals.

### 2.5 Data analysis

**Figure 1:**
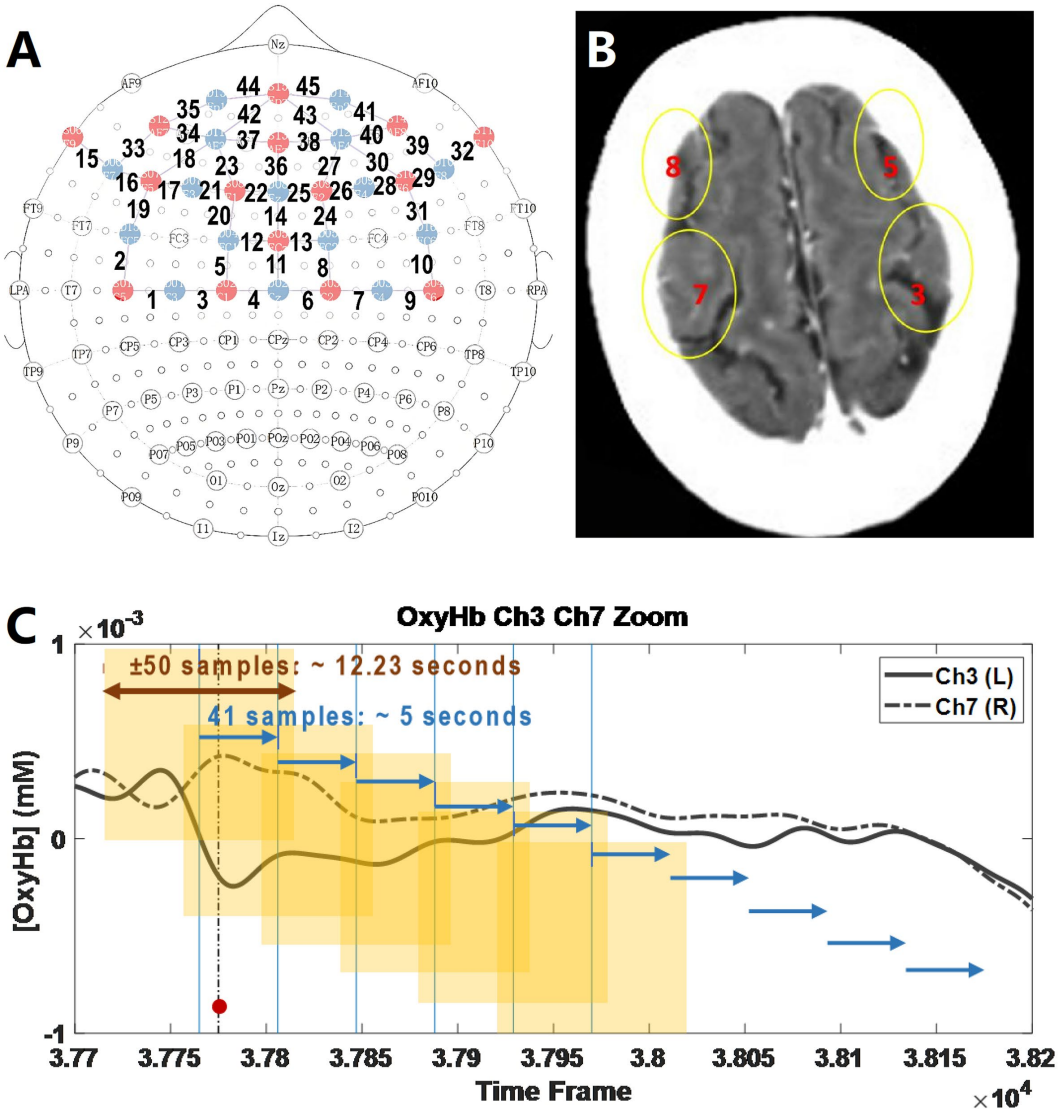
Overview of fNIRS data acquisition and analysis. **(A)** Site map of fNIRs records. Signals of forty-five fNIRS channels on the patient were recorded. The locations of the channels were intended to record the signals of the territory of MCA near the cortices. Moreover, channels located at the cortices of other arteries’ territories were recorded as references. **(B)** Blood supply areas in monitoring. **(C)** fNIRS data analysis. Example of the time windows for calculating the standard deviation (STD). The yellow highlighted zone represents the time windows and the blue arrows show the sliding speed. The standard deviation (STD) of the signals crossing these 12.23 s (±50 samples) was computed, representing the stability of the signals. A concise procedural timeline is provided in the text and legend to illustrate the alignment between balloon inflation/deflation, stent manipulation, and the corresponding fNIRS analysis windows.

Signals from 45 channels were analyzed to assess regional oxygen saturation (rSO2) in the parietal, temporal, and frontal cortices (Figure 1A). Four regions of interest (ROIs 3, 5, 7, 8; Figure 1B), located near the precentral and postcentral gyri and thus related to motor and sensory functions, were selected for detailed analysis. Changes in oxyhemoglobin concentration (△[OxyHb]) at the affected site and its contralateral homologous site were calculated. To quantify fluctuations, the standard deviation (STD) of △[OxyHb] was computed at each time window. A time window of ±50 samples (12.29 s) was applied to calculate the STD. The time windows slide with a speed of 41 samples, meaning around 5 s, as shown in Figure 1C.

## 3 Case series description

For clarity, the temporal sequence of procedural steps (balloon inflation, balloon deflation, stent deployment, and stent release) is described alongside the corresponding fNIRS analysis windows, allowing readers to interpret the signal fluctuations in relation to intraoperative events.

### 3.1 Case 1

**Figure 2:**
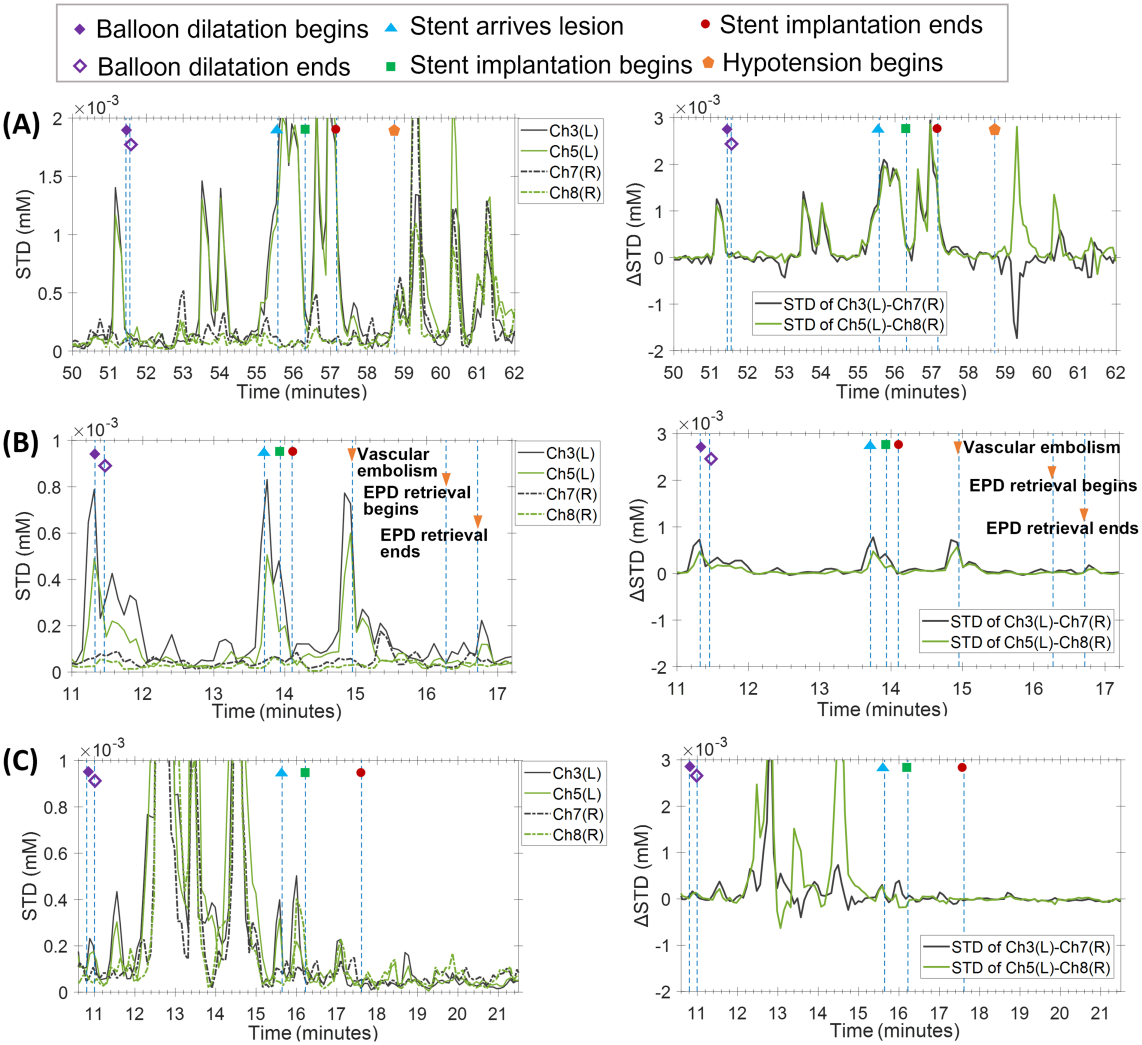
fNIRS dynamic recordings during left carotid artery stenting (Case 1–3). **(A)** Carotid sinus syndrome: the patient showed increased local oxygen saturation standard deviation (SD) before and after balloon dilatation and during stent implantation. Severe carotid sinus syndrome occurred 2 min after stent placement, with corresponding SD increase. Similar patterns were observed in the contralateral side. **(B)** Embolic event: the patient exhibited increased local SD before and after balloon dilatation and during stent placement. Emboli trapped in the protection device led to transient SD increase, which resolved after removal. Similar contralateral changes were noted. **(C)** Transient hypotension: the patient experienced transient hypotension after stent implantation. Local SD increased during balloon dilatation and stent placement, with additional fluctuation likely due to patient agitation. Contralateral SD changes were similar.

Approximately 2 min after stent deployment, the patient developed abrupt hypotensive shock (systolic BP fell from ~140 mmHg to ~80 mmHg within seconds). This was immediately followed by reduced consciousness, global aphasia, and right-sided weakness, with the patient unable to follow simple commands. After vasopressor administration, blood pressure normalized within 3–4 min, and neurological deficits began to improve shortly thereafter, with consciousness recovering first and mild aphasia persisting the longest. fNIRS demonstrated a progressive rise in STD of *Δ*[OxyHb] beginning shortly after angioplasty and peaking around the onset of shock, mirroring the temporal evolution of the clinical event (Figure 2A).

### 3.2 Case 2

Immediately following stent release, angiography detected distal embolization within the protection device, despite stable systemic blood pressure. Within 1–2 min, the patient developed acute right hemiplegia and partial expressive aphasia, both reaching maximal severity rapidly. Intravenous tirofiban was administered, and neurological deficits improved gradually over 48 h, with motor function recovering earlier than speech. fNIRS revealed a pronounced STD surge immediately after stenting, followed by a second, distinct peak at the time of protection device retrieval, aligning precisely with embolic manipulation and symptom onset (Figure 2B).

### 3.3 Case 3

During post-stent angiography, the patient experienced transient asymptomatic hypotension (approximately 30% systolic BP drop), which resolved spontaneously within less than 1 min. No neurological deficits occurred throughout the procedure. Nevertheless, fNIRS recorded a clear, localized increase in STD of *Δ*[OxyHb] immediately following stent deployment, suggesting sensitivity to subtle hemodynamic fluctuations even in the absence of clinical symptoms (Figure 2C).

### 3.4 Case 4

**Figure 3:**
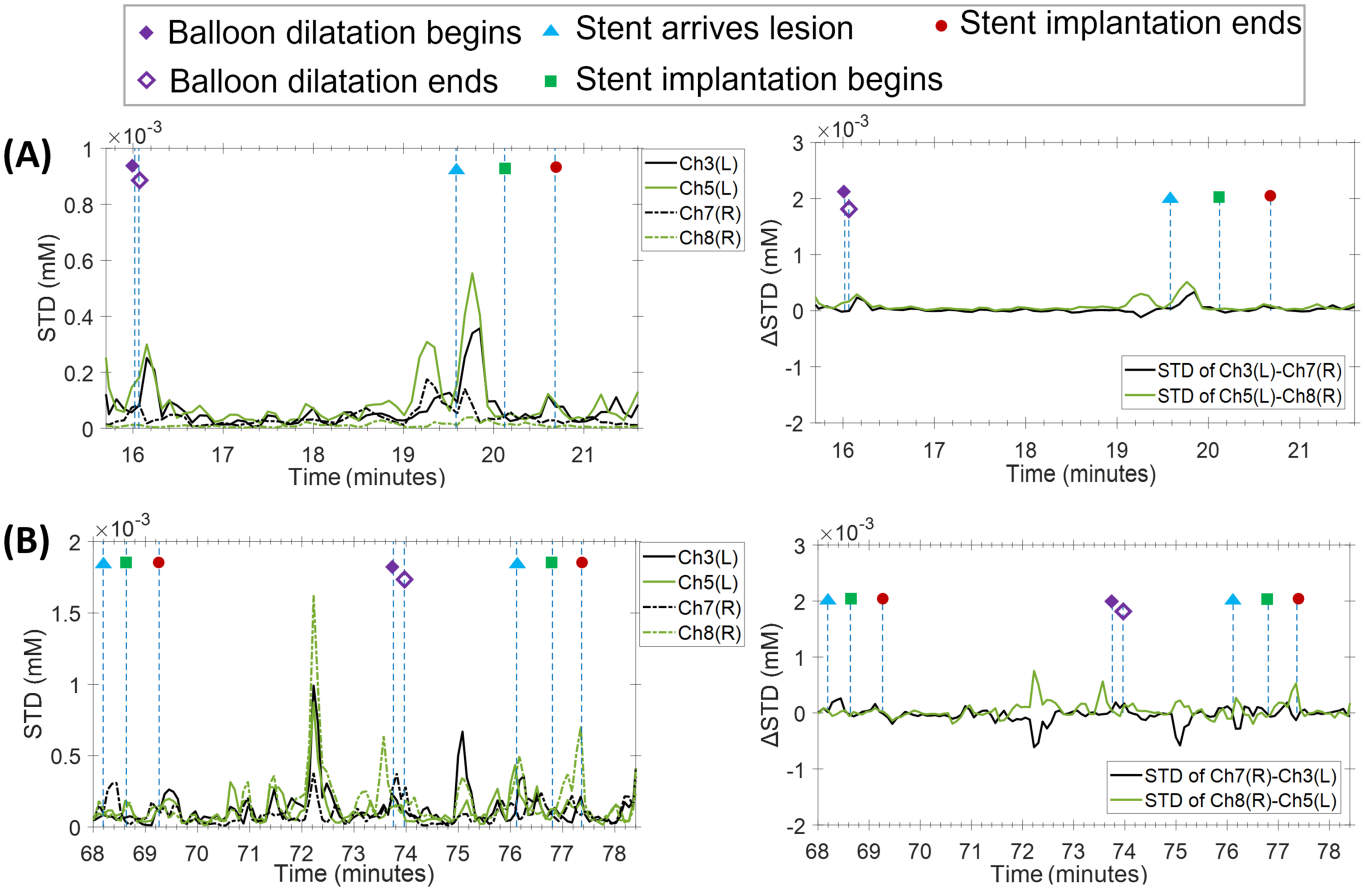
fNIRS dynamic recordings during carotid artery stenting (Case 4, 5). **(A)** No intraoperative complications (left-sided): the patient tolerated the procedure well, showing increased local SD before and after balloon dilatation and during stent implantation. Contralateral SD changes were similar. **(B)** Hypotension (right-sided): the patient showed increased local SD before and after balloon dilatation and during stent placement. Contralateral SD changes were similar.

Hemodynamics remained stable throughout the procedure, with no hypotension, bradycardia, or neurological symptoms. However, fNIRS still showed a brief, event-locked elevation in STD of Δ[OxyHb] at the moment of stent deployment, indicating a reproducible cortical oxygenation response to mechanical intervention itself rather than systemic physiological changes (Figure 3A).

### 3.5 Case 5

During multilevel stenting, the patient experienced procedural hypotension (systolic BP decrease of ~25%), which responded promptly to vasopressor administration and returned to baseline within 2–3 min. No neurological deficits were reported at any time. fNIRS demonstrated increased variability in both STD and mean *Δ*[OxyHb] immediately after stent placement, followed by gradual stabilization as blood pressure normalized, reflecting the short-lived hemodynamic instability (Figure 3B).

## 4 Discussion

This study explores the feasibility of using multi-channel fNIRS to monitor cortical oxygenation during CAS procedures. Our findings demonstrate that fNIRS can detect dynamic changes in cerebral oxygenation induced by hemodynamic fluctuations, such as carotid sinus stimulation and distal embolic events, providing real-time insight into perioperative cerebral perfusion.

In all five cases, CAS was technically successful, with residual stenosis less than 30%. fNIRS monitoring consistently showed transient increases in the variability (STD) of *Δ*[OxyHb] following angioplasty or stent deployment, reflecting momentary hemodynamic perturbations associated with vessel opening. Notably, transient reductions in cortical oxygenation were observed in patients experiencing hypotension or bradycardia related to carotid sinus reflex. In one patient, focal neurological deficits—including hemiplegia, aphasia, and reduced consciousness—coincided with a decline in cortical oxygenation. These symptoms gradually resolved as blood pressure normalized, illustrating that fNIRS can provide early warning of hemodynamically mediated cerebral hypoperfusion. Although causality between blood pressure changes and neurological symptoms cannot be definitively established, the temporal correlation supports the utility of fNIRS for real-time monitoring. Notably, inspection of the raw light-intensity traces did not reveal motion-induced discontinuities, and the observed STD elevations occurred consistently in temporal synchrony with procedural events. This pattern supports a physiologic rather than artifactual origin of the variability changes.

Previous studies have mainly applied dual-channel NIRS focused on the frontal region, limiting the ability to capture regional heterogeneity of cortical oxygenation (McCleary et al., 1998; Nakagawa et al., 2021; Kakumoto et al., 2018). For example, A. J. McCleary used dual-channel NIRS combined with transcranial Doppler (TCD) to monitor rSO₂ during CAS under general anesthesia. While reductions in oxygenation were detected during balloon inflation and reperfusion hyperemia was observed post-stent, single-site monitoring could not reflect lateral differences or detect distal embolic events, and complications such as intrastent thrombosis posed high risks (McCleary et al., 1998). Similarly, Ichiro Nakagawa reported fNIRS detection of thromboembolic events in a subset of CAS patients, but the monitoring was limited to 1–2 frontal sites, leaving many cortical areas unassessed (Nakagawa et al., 2021).

Our study improves upon these limitations by employing multi-channel fNIRS, covering 45 cortical sites across the anterior circulation. This approach allows for detection of spatially heterogeneous alterations in cortical oxygenation that may arise from localized hypoperfusion or embolization. Compared with traditional dual-channel NIRS, which is typically positioned on the forehead and thus reflects hemodynamics from only a small portion of the anterior circulation, multi-channel fNIRS provides several clear advantages for intraoperative monitoring during CAS. First, the flexible optode arrangement enables coverage of bilateral frontal, temporal, and parietal cortices, including regions more directly supplied by the treated internal carotid artery. This broader spatial sampling allows detection of lateralized or region-specific changes that may be missed by single-site frontal monitoring. Second, when channels are placed symmetrically across hemispheres, multi-channel recordings permit direct comparison of hemodynamic responses between the affected and unaffected sides, thereby improving the ability to identify focal cortical disturbances associated with hypotension or embolization. Third, increased spatial resolution enables assessment of cortical areas distal to the stenosis, which may show early perfusion instability even in the absence of systemic hemodynamic changes. Finally, multi-channel data allow exploratory analyses such as cross-regional temporal correlations or functional-connectivity patterns, which may provide insight into network-level responses to carotid manipulation and help refine future cerebroprotection strategies. In one patient, distal plaque rupture led to vascular occlusion at the site of the protection device. fNIRS demonstrated a pronounced transient fluctuation in *Δ*[OxyHb] signals at the time of distal embolization, reflected by a sharp rise in STD, which subsided after restoration of distal flow. This demonstrates that multi-channel fNIRS can capture dynamic and region-specific changes in cerebral perfusion that single- or dual-channel systems might miss.

In addition, transient hypotension and bradycardia are well-documented during CAS due to carotid sinus reflex activation (McKevitt et al., 2003; Chung et al., 2012; Ogasawara et al., 2007). In our cohort, one patient developed hypotensive shock approximately 2 min after stent release. Cortical oxygenation decreased shortly after stent deployment, preceding overt neurological symptoms. This suggests that fNIRS monitoring may provide earlier detection of cerebral hypoperfusion than clinical observation alone, complementing non-invasive blood pressure monitoring (Rodriguez et al., 2006).

Despite these advantages, fNIRS is not without limitations. Measurement can be affected by extracranial factors, including scalp blood flow, sensor movement, and ambient light interference (Kurth and Uher, 1997). In particular, head movement and hair coverage can introduce significant signal artifacts, limiting data quality. While cortical oxygenation reflects relative changes in cerebral perfusion, absolute quantification remains challenging, and fNIRS cannot fully substitute for structural imaging or direct cerebral blood flow measurements. Nevertheless, in our study, all patients had arterial oxygen saturation >95%, minimal blood loss, and brief occlusion times, suggesting that major extracranial confounders were unlikely to significantly impact observed cortical oxygenation changes.

Another limitation relates to the intrinsic variability of fNIRS measurements. Physiological oscillations—including cardiac pulsation (~1–1.5 Hz), respiration (~0.2–0.4 Hz), and Mayer waves (~0.1 Hz)—can introduce fluctuations unrelated to the CAS procedure. These components were minimized in our analysis by applying a 0.01–0.2 Hz band-pass filter. Extracranial hemodynamics, particularly scalp blood flow, also contribute to optical signals and cannot be completely separated from cortical activity in the absence of short-separation channels. Although synchronized blood pressure recordings helped contextualize fNIRS fluctuations and no motion-related artifacts were detected, future studies incorporating short-channel regression and multimodal physiological monitoring will further improve signal specificity. Despite these sources of variability, the temporal alignment between procedural events and STD elevations supports the validity of our main findings.

Transient cerebral hemodynamic perturbations during CAS, including hypoperfusion, hyperperfusion, and brief reperfusion episodes, are known to influence BBB integrity through endothelial and neurovascular coupling mechanisms. The multi-channel fNIRS system, by capturing spatially resolved cortical oxygenation dynamics, may serve as a surrogate marker for these BBB-related physiological fluctuations. Integrating fNIRS-based monitoring with biomarkers or imaging indicators of BBB permeability could therefore bridge real-time intraoperative physiology with mechanistic cerebroprotection research. This aligns with emerging translational efforts to preserve BBB function as a key target for improving stroke treatment efficacy.

In summary, multi-channel fNIRS provides a sensitive, non-invasive method for real-time monitoring of cerebral oxygenation during CAS, capable of detecting hemodynamic disturbances and focal hypoperfusion events. Compared with previous dual-channel or frontal-limited NIRS studies, our approach captures spatial heterogeneity and temporal dynamics more comprehensively, highlighting its potential clinical value in guiding intraoperative management and early detection of complications. Future studies with larger cohorts are warranted to validate these findings and further define fNIRS thresholds predictive of neurological events.

## Data Availability

The raw data supporting the conclusions of this article will be made available by the authors, without undue reservation.
